# Molecular and Physiological Alterations in Chickpea under Elevated CO_2_ Concentrations

**DOI:** 10.1093/pcp/pcaa077

**Published:** 2020-06-05

**Authors:** Paramita Palit, Raju Ghosh, Priya Tolani, Avijit Tarafdar, Annapurna Chitikineni, Prasad Bajaj, Mamta Sharma, Himabindu Kudapa, Rajeev K Varshney

**Affiliations:** International Crops Research Institute for the Semi-Arid Tropics (ICRISAT), Patancheru 502324, India

**Keywords:** Climate change, Differentially expressed genes, Elevated CO_2_ concentration, RNA-Seq, Stress pathways, Transcriptome

## Abstract

The present study reports profiling of the elevated carbon dioxide (CO_2_) concentration responsive global transcriptome in chickpea, along with a combinatorial approach for exploring interlinks between physiological and transcriptional changes, important for the climate change scenario. Various physiological parameters were recorded in two chickpea cultivars (JG 11 and KAK 2) grown in open top chambers under ambient [380 parts per million (ppm)] and two stressed/elevated CO_2_ concentrations (550 and 700 ppm), at different stages of plant growth. The elevated CO_2_ concentrations altered shoot and root length, nodulation (number of nodules), total chlorophyll content and nitrogen balance index, significantly. RNA-Seq from 12 tissues representing vegetative and reproductive growth stages of both cultivars under ambient and elevated CO_2_ concentrations identified 18,644 differentially expressed genes including 9,687 transcription factors (TF). The differential regulations in genes, gene networks and quantitative real-time polymerase chain reaction (qRT-PCR) -derived expression dynamics of stress-responsive TFs were observed in both cultivars studied. A total of 138 pathways, mainly involved in sugar/starch metabolism, chlorophyll and secondary metabolites biosynthesis, deciphered the crosstalk operating behind the responses of chickpea to elevated CO_2_ concentration.

## Introduction

Concentrations of carbon dioxide (CO_2_) in the atmosphere surged at a record-breaking speed of 411 parts per million (ppm) in 2019 (https://www.co2.earth/). The unprecedented rise in atmospheric CO_2_ concentration strengthens the uncertainty of altered crop yield and other physiological parameters. The Intergovernmental Panel on Climate Change 2018 special report on the ‘impact of global warming of 1.5°C above pre-industrial levels’ pledged to limit global warming to 1.5°C. This requires ‘CO_2_ emissions to fall 45% from 2010 levels by 2030, and reaching ‘‘net zero” around 2050’ (Masson-Delmotte et al. 2018). Hence, the major key player of changing climate will be the new abiotic stress associated with elevated CO_2_ concentration. The popular notion of association of increment in plant yield with elevated CO_2_ concentration is often highly compromised by a significantly lowered biomass and grain production in crops (Battisti and Naylor 2009). This drastic alteration in the surge of primary photosynthates of plants will profoundly influence the physiological parameters. Hence, elevated CO_2_ can be looked upon as a previously unseen new stress response for plants, which they are unable to adapt to yet. As the amount of CO_2_ intake in plants affects the carbon partitioning, it affects the overall plant health and plant stress mechanism as evidenced by physiological studies (Rai et al. 2016). Thus, in contrast to other abiotic and biotic stresses, plants likely have not evolved a specific mechanism for responding to elevated CO_2_. This will lead to a nonspecific, dramatic response as the plants try to find a way to deal with this significant stress (Liu 2013). There have been a few recent reports on the model system *Arabidopsis* (Caldera et al. 2017), wheat (Tian et al. 2013) and maize (Ge et al. 2018) using Open Top Chambers (OTCs) to explore the molecular mechanism under elevated CO_2_. Apart from these, investigations are mostly limited to studying plant growth and physiological parameters separately (Körner 2006). Moreover, these reports were not coherent studies that included both physiological and molecular alterations. A number of metabolic and proteomic studies have recently shown the alteration in various metabolic processes or pathways, such as photosynthetic carbon fixation, respiratory metabolism, cellular growth and stress defense under elevated CO_2_ (Yu et al. 2017). Needless to say, these alterations are often preceded by some changed physiological parameters.

Legumes, the ‘plant meat’, are an excellent source of proteins and, thus, represent a timely answer to malnutrition in achieving food security goals (Röös et al. 2020). Chickpea (*Cicer arietinum*) is the second most widely grown legume crop after soybean. It plays a crucial role in food security in developing countries particularly in semi-arid regions (Kudapa et al. 2018). The availability of the chickpea genome sequence (Varshney et al. 2013) and a perusal of the literature exploring transcriptome changes, including the chickpea gene expression atlas (Kudapa et al. 2018), small RNA and degradome profiling (Garg et al. 2019), have crowned it as an ideal/important legume and semi-arid crop to study functional genomics under elevated CO_2_.

Altered gene expression patterns and transcriptome changes to combat stress response are two important strategies of plants. The potential of next-generation sequencing (NGS)-based technologies in unraveling the global transcriptome changes is a convenient and rapid method to study gene expression. It can be further used to predict putative gene function (Kudapa et al. 2014). A plethora of literature is currently available in chickpea transcriptomics under various developmental and stress conditions (Afonso-Grunz et al. 2014, Kudapa et al. 2014). Recently, a number of transcriptome studies on the effects of elevated CO_2_ have been reported in Arabidopsis (Niu et al. 2016), maize (Ge et al. 2018), etc. However, to the best of our knowledge, no such study deciphering the global gene expression changes correlating with physiological and molecular changes under elevated CO_2_ has been undertaken in legumes so far.

The present study deciphered the physiological alterations in chickpea at different growth stages associated with the genome-wide transcriptome changes under ambient (380 ppm) and elevated CO_2_ (550 ppm as moderate and 700 ppm as high level of stress) concentrations. By considering the physiological alterations and the genome-wide transcriptome changes, this acts as an effective strategy to identify the candidate gene pool and key players of elevated CO_2_ concentration-mediated stress-responsive pathways. This approach should be helpful to develop climate-resilient chickpea varieties in the future.

## Results

Elevated CO_2_ concentration affects plant growth and photosynthetic capacity. Important and general physiological parameters, such as shoot and root length, nodulation, total chlorophyll, total flavonoid contents and the nitrogen balance index (NBI), were chosen for taking observations in the study. Physiological alterations together with transcriptome changes in two chickpea cultivars—namely JG 11, a representative of *desi* type, and KAK 2, a representative of *kabuli* type—under ambient (380 ppm) and elevated CO_2_ concentrations (550 and 700 ppm) at different growth stages progressed from 15 d (vegetative), 30 d (reproductive) and 45 d (maturity) were studied. The selected cultivars are different in many phenotypic and seed features (Wood et al. 2011). It was an important quest to see whether they reacted differentially to elevated CO_2_ concentrations, which can largely affect performance. Physiological parameters, root and shoot lengths, nodulation and transcriptome changes were studied up to reproductive stages, while the rest of the physiological parameters (total chlorophyll, total flavonoid contents and NBI) were studied up to maturity. The experimental pipeline followed in this study is summarized in Supplementary Fig. S1.

### Effect of elevated CO_2_ concentrations on physiological parameters

#### Root and shoot lengths

**Fig. 1:**
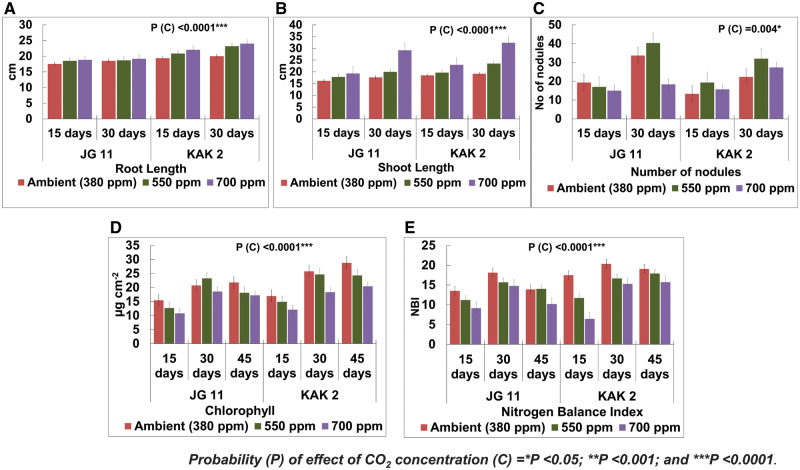
Effect of elevated CO_2_ concentrations on physiological parameters. (A) Shoot length in chickpea (JG 11 and KAK 2) raised under different CO_2_ concentrations—ambient (380 ppm) and elevated (550 and 700 ppm) CO_2_. (B) Root length in chickpea (JG 11 and KAK 2) raised under different CO_2_ concentrations—ambient (380 ppm) and elevated (550 and 700 ppm) CO_2_. (C) Number of nodules in chickpea (JG 11 and KAK 2) raised under different CO_2_ concentrations—ambient (380 ppm) and elevated (550 and 700 ppm) CO_2_. (D) Total chlorophyll content in chickpea (JG 11 and KAK 2) raised under different CO_2_ concentrations—ambient (380 ppm) and elevated (550 and 700 ppm) CO_2_. (E) NBI in chickpea (JG 11 and KAK 2) raised under different CO_2_ concentrations—ambient (380 ppm) and elevated (550 and 700 ppm) CO_2_.

The effects of elevated CO_2_ concentrations on root and shoot lengths were evaluated using a three-way analysis of variation (ANOVA). The probability (*P*-value) of the factors, different days (D), cultivar/genotypes (G) and CO_2_ interaction (C) for root length were *P* = 0.002, *P* < 0.0001 and *P* < 0.0001, respectively, which were statistically significant.The probability of all three factors, such as different days (D), cultivar/genotypes (G) and CO_2_ interaction (C), for shoot length was statistically significant with *P* < 0.0001. At maturity, chickpea plants became lanky, hence shoot and root lengths were tabulated up to 30 d (reproductive stage of the plant). The shoot and root lengths of both the cultivars, JG 11 and KAK 2, were enhanced with elevated CO_2_ concentrations (550 and 700 ppm) as compared to controls (380 ppm, which was ambient CO_2_ concentration) (Fig. 1A, B and Supplementary Table S1).

#### Root nodulation (number of nodules)

Effect of elevated CO_2_ concentrations on the nodulation number was evaluated using three-way ANOVA.The probability of the factor CO_2_ concentration (C) was *P* = 0.004 and that of different days (D) was *P* < 0.0001, which were statistically significant. In both the cultivars, the number of nodules was increased at 550 ppm at the reproductive stage (30 d). In JG 11, at the reproductive stage (30 d), the average nodule number in ambient CO_2_ concentration (380 ppm) was 33.67. This was increased to 40.33 at 550 ppm. Similarly, in KAK 2, the average number of nodules was 22.33 and increased to 32.00 at 550 ppm at the reproductive stage. In the case of JG 11 at 700 ppm, the average number of nodules decreased to 18.33. However, in the case of KAK 2, an opposite trend was observed at 700 ppm. The average number of nodules increased from 22.33 at 380 ppm to 27.33 at 700 ppm (Fig. 1C and Supplementary Table S1).

#### Total chlorophyll content

Effect of elevated CO_2_ concentrations on the total chlorophyll content and NBI was also evaluated using a three-way ANOVA. In the case of chlorophyll content, the probability of the factors, different days (D), cultivar/genotypes (G) and CO_2_ interaction (C) was *P* < 0.0001, which was statistically significant (Fig. 1D and Supplementary Table S1). For both the cultivars, the total chlorophyll content was decreased with increased CO_2_ concentration. For example, in JG 11, the total chlorophyll content was decreased from 15.43 to 12.70 and 10.80 µg cm^−2^ with respect to different CO_2_ concentrations viz. ambient (380 ppm), 550 and 700 ppm at vegetative stage, respectively. At the reproductive stage, a slight increase in chlorophyll content was observed at 550 ppm. The total chlorophyll content varied with respect to elevated CO_2_ concentrations and the plant age for both cultivars. In the case of JG 11, under ambient CO_2_ concentration (380 ppm), the total chlorophyll content was measured to be 15.43 µg cm^−2^ at 15 d. This was increased to 20.73 and 21.77 µg cm^−2^ as the plant age-progressed from reproductive stage to maturity, whereas, in the case of KAK 2, it got increased from 16.97 to 25.77 and 28.83 µg cm^−2^ as the plant age-progressed from vegetative stage to reproductive stage and maturity, respectively (Supplementary Table S1).

#### Total flavonoid content and NBI

In the case of flavonoid content, no significant difference was observed under elevated CO_2_ concentrations (Supplementary Table S1). In the case of NBI, the probability of the factors, such as different days (D), cultivar/genotypes (G) and CO_2_ interaction (C), was statistically significant with *P* < 0.0001, *P* = 0.04 and *P* < 0.0001, respectively. The NBI was decreased with increased elevated CO_2_ concentrations (Fig. 1E and Supplementary Table S1).

### Transcriptome analysis for the identification of differentially expressed genes

Transcriptome analysis through an RNA-Seq approach was carried out using 12 samples harvested at different stages of plant growth and concentrations of elevated CO_2_ (Table 1). A total of 605 million (M) raw reads were generated and subjected to quality control (QC) filtering resulted in 491 M (90.7%) filtered reads.The details of RNA-Seq samples along with the reads, alignment and mapping data statistics are tabulated in Table 2.

**Table 1:** Details of the samples harvested under different CO_2_ concentrations for RNA-Seq

Sl. no.	Sample ID	Tissue	CO_2_ concentrations	Cultivar	Stage (days old)
1	J-380-15	The shoot, leaves and apical meristem	380 ppm (control)	JG 11	15
2	J-380-30				30
3	K-380-15			KAK 2	15
4	K-380-30				30
5	J-550-15		550 ppm (stress)	JG 11	15
6	J-550-30				30
7	K-550-15			KAK 2	15
8	K-550-30				30
9	J-700-15		700 ppm (stress)	JG 11	15
10	J-700-30				30
11	K-700-15			KAK 2	15
12	K-700-30				30

**Table 2:** Summary of RNA-Seq data generated, reads alignment and mapping statistics

Sample ID	CO_2_ concentration	Age (days)	Total raw reads (million)	Total filtered reads (million)	Aligned reads (million)	% alignment
J-380-15	380 ppm (control)	15	48.8	39.0	35.5	91.02
J-380-30		30	51.1	41.4	37.7	91.06
J-550-15	550 ppm (stress)	15	45.4	36.3	32.5	89.53
J-550-30		30	45.3	36.9	33.7	91.32
J-700-15	700 ppm (stress)	15	51.4	41.9	38.2	91.16
J-700-30		30	49.3	40.0	36.2	90.50
K-380-15	380 ppm (control)	15	48.3	39.3	35.8	91.19
K-380-30		30	52.6	42.6	37.6	88.26
K-550-15	550 ppm (stress)	15	57.9	46.8	42.6	91.02
K-550-30		30	52.9	43.8	40.0	91.32
K-700-15	700 ppm (stress)	15	50.6	40.7	37.1	91.15
K-700-30		30	52.1	42.7	37.8	88.52

These 491 M filtered reads were used for further downstream analysis as mentioned in the pipeline (Supplementary Fig. S1). The sequencing reads in each sample varied from 36.3 to 46.8 M filtered reads. The sample IDs were denoted by the cultivar name (JG 11 or KAK 2), CO_2_ concentration (380, 550 or 700 ppm) imposed and stage of the plant (15 or 30 d). For example, K-700-30 denoted KAK 2 at 700 ppm and at the reproductive stage (30 d). Hereafter, the samples are represented with sample IDs. Mapping of the 491 M filtered reads to the chickpea genome (Varshney et al. 2013) resulted in the alignment of 444 M (90.5%) reads ranging from 88.3% (K-380-30) to 91.3% (J-550-30 and K-550-30) (Table 2). For studying the differential gene expression, the genes with very low expression values in all the samples were filtered out. A gene was considered to be expressed in a given sample if its FPKM (fragments per kilobase of transcript per million mapped reads) was ≥1, and the quantification status was ‘OK’. Using these criteria, a total of 19,278 genes were expressed in at least one of the samples.

**Fig. 2:**
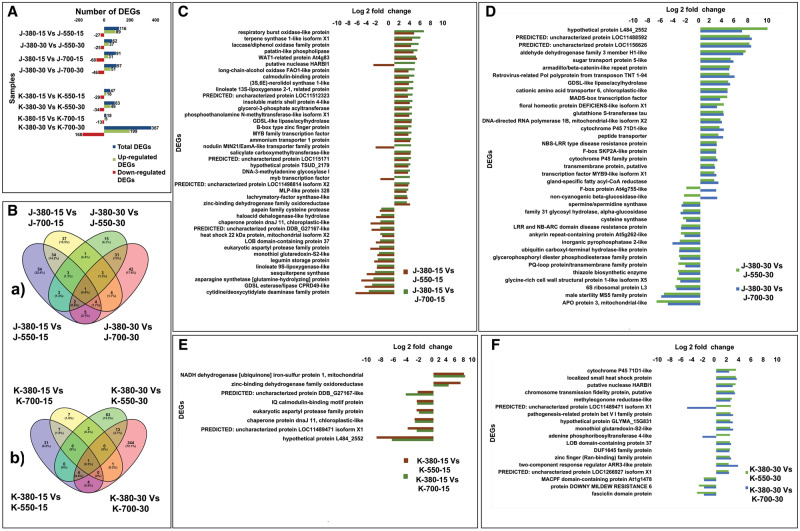
Graphical representation of significantly DEGs in various samples. (A) Distribution of significant DEGs of JG 11 (J) and KAK 2 (K) varieties at 380 (control), 550 (stressed) and 700 ppm (stressed) of CO_2_ concentrations at vegetative (15 d) and reproductive (30 d) stages. (B) Venn diagram showing cultivar-specific distribution of significant DEGs at 380 (control), 550 (stressed) and 700 ppm (stressed) of CO_2_ concentration at vegetative (15 d) and reproductive (30 d) stages. (a) Common and exclusive DEGs in JG 11 under 550 (stressed) and 700 ppm (stressed) of CO_2_ concentration over 380 ppm (control) and (b) common and exclusive DEGs in KAK 2 under 550 (stressed) and 700 ppm (stressed) of CO_2_ concentration over 380 ppm (control). [The sample designation as follows: J-380-15 vs J-550-15 = number of DEGs at 550 ppm over 380 ppm CO_2_ concentration at the vegetative stage (15 d) of JG 11; J-380-30 vs J-550-30 = number of DEGs at 550 ppm over 380 ppm CO_2_ concentration at the reproductive stage (30 d) of JG 11; J-380-15 vs J-700-15 = number of DEGs at 700 ppm over 380 ppm CO_2_ concentration at the vegetative stage (15 d) of JG 11; and J-380-30 vs J-700-30 = number of DEGs at 700 ppm over 380 ppm CO_2_ concentration at the reproductive stage (30 d) of JG 11] (similar designation for K = KAK 2). (C) Fold change of CO_2_ responsive common DEGs between 550 and 700 ppm over control (380 ppm) in JG 11 variety at the vegetative stage (15 d). (D) Fold change of CO_2_ responsive common DEGs between 550 and 700 ppm over control (380 ppm) in JG 11 variety at the reproductive stage (30 d). (E) Fold change of CO_2_ responsive common DEGs between 550 and 700 ppm over control (380 ppm) in KAK 2 variety at the vegetative stage (15 d). (F) Fold change of CO_2_ responsive common DEGs between 550 and 700 ppm over control (380 ppm) in KAK 2 variety at the reproductive stage (30 d).

The total number of significantly differentially expressed genes (DEGs) in various samples has been graphically represented in Fig. 2A. The Venn diagram illustrates the common and exclusive set of DEGs with respect to cultivar, stage and stress imposition (elevated CO_2_ concentration) (Fig. 2B). The cultivar-specific regulation of common DEGs at 550 and 700 ppm elevated CO_2_ concentrations is graphically represented in Fig. 2C–F. The aforesaid differential physiological responses were further correlated with the altered dynamics of both cultivar- and stage-specific DEGs.

#### Distribution of DEGs in chickpea cultivars under stress conditions over control

The fold changes for DEGs under both 550 and 700 ppm elevated CO_2_ concentrations were calculated over that of the ambient (control), i.e. 380 ppm elevated CO_2_ concentration for each cultivar. A total of 10,464 regulated DEGs were identified (Supplementary Table S2).

#### Growth stage-specific response of DEGs

In JG 11, a total of 116 (89 upregulated and 27 downregulated) and 62 (37 upregulated and 25 downregulated) DEGs were differentially regulated in J-550-15 and J-550-30, respectively, whereas a total of 91 (31 upregulated and 60 downregulated) and 97 (51 upregulated and 46 downregulated) DEGs were differentially regulated in J-700-15 and J-700-30, respectively.Interestingly, at the vegetative stage, more genes were differentially regulated at 550 ppm elevated CO_2_ concentration than at 700 ppm elevated CO_2_ concentration, whereas, at the reproductive stage (30 d), more genes were differentially regulated at 700 ppm elevated CO_2_ concentration (Fig. 2A). In KAK 2, a total of 47 (18 upregulated and 29 downregulated) and 83 (49 upregulated and 34 downregulated) DEGs were differentially regulated in between K-550-15 and K-550-30, respectively, whereas, in K-700-15 and K-700-30, 18 (5 upregulated and 13 downregulated) and 367 (199 upregulated and 168 downregulated) DEGs were differentially regulated. An exceptionally higher number of DEGs were expressed at the reproductive stage under 700 ppm elevated CO_2_ concentration than that of the other samples (Fig. 2A). A number of growth stage-specific DEGs were upregulated in JG 11 like respiratory burst oxidase-like protein, terpene synthase 1-like isoform X1 and laccase/diphenol oxidase family protein, whereas cytidine/deoxycytidylate deaminase family protein and GDSL esterase/lipase CPRD49-like protein were highly downregulated proteins. At the reproductive stage, the highly upregulated DEGs in J-550-30 and J-700-30 were the Hypothetical protein L484_2552 showing 9.65- and 5.99-fold upregulation, respectively. Uncharacterized proteins LOC11488592 and LOC1156626 showed more than 7-fold upregulation in both stages. Similarly, aldehyde dehydrogenase family 3 member H1-like, sugar transport protein 5-like, MADS-box transcription factor (TF), glutathione *S*-transferase tau and NBS-LRR type disease resistance proteins were some of the important DEGs that were upregulated at the reproductive stage under both 550 and 700 ppm elevated CO_2_ concentrations. Likewise, the genes APO protein 3, mitochondrial-like, male sterility MS5 family protein and inorganic pyrophosphatase 2-like protein were downregulated at both 550 and 700 ppm at the reproductive stage. Interestingly, F box protein At4g755-like (−2 and 2 folds) and non-cyanogenic beta-glucosidase like protein (−2.3 and 2.4 folds) showed reciprocal regulation at 550 and 700 ppm elevated CO_2_ concentrations, respectively. These observations further confirmed that the plant growth stage plays an important role in modulating response to elevated CO_2_ concentrations in chickpea.

#### Cultivar-specific response of DEGs

The distribution of common and exclusively expressed DEGs in both cultivars at two different growth stages and two different elevated CO_2_ concentrations has been separately depicted in two Venn diagrams (Fig. 2B). These Venn diagrams clearly indicate that the number of significantly regulated DEGs was much higher in KAK 2 at the reproductive stage, especially under 700 ppm elevated CO_2_ concentration. An exclusive set of 344 DEGs was found expressed at K-700-30, which was absent in other stages especially under the 550 ppm elevated CO_2_ concentration stress point (Fig. 2B). This further evidenced the notion of cultivar-specific responses found in physiological parameters governing CO_2_ concentration-mediated stress especially at the reproductive phase of chickpea.

#### Elevated CO_2_ concentration-specific response of DEGs

The imposed elevated CO_2_ concentration played a critical role in showing differential response in the cultivars at a particular growth stage. The fold regulation of elevated CO_2_ concentration responsive common DEGs in JG 11 at the vegetative and reproductive stages is shown graphically in Fig. 2C, D. The same in KAK 2 is presented in Fig. 2E, F. Most of the DEGs showed similar patterns of regulation with the increased degree of elevated CO_2_ concentrations. A total of five DEGs at the vegetative stage in JG 11 showed opposite/reciprocal regulation. These were three DEGs between J-550-15 and J-700-15, namely an MYB TF, a nodulin MtN21/EamA-like transporter family protein and a putative nuclease HARBI1, and two DEGs between J-550-30 and J-700-30, namely an F box protein At4g755-like and non-cyanogenic beta-glucosidase-like protein, respectively. For example, in J-550-15, MYB TF and nodulin MtN21/EamA-like transporter family protein showed a 2.6- and 2.8-fold upregulation, respectively. However, at J-700-15, it showed a −2.3- and −3.1-fold downregulation. In the case of KAK 2, 83 and 367 DEGs were regulated at K-550-30 and K-700-30, respectively. However, in the case of K-700-30, a much higher number of DEGs were altered. This denoted that the interaction between elevated CO_2_ concentrations and chickpea largely depended on the degree or amount of stress imposed. A total of eight DEGs were common and significantly upregulated or downregulated between K-550-15 and K-700-15 (Fig. 2E). NADH dehydrogenase (ubiquinone) iron-sulfur protein 1, mitochondrial (4.66- and 4.8-fold) and zinc-binding dehydrogenase family oxidoreductase were (4.1- and 2.31-fold) upregulated DEGs in those samples. Interestingly, hypothetical protein L484_2552, which showed the highest downregulation at the vegetative stage, had a high cultivar-specific expression pattern. Only 18 DEGs were common among significantly regulated DEGs between K-550-30 and K-700-30 (Fig. 2F). Among them, cytochrome P45 71D1-like protein (3.31- and 2.16-fold), localized small heat shock protein (3.28- and 3.4-fold), putative nuclease HARBI1 (3.23- and 2.75-fold), pathogenesis-related protein bet VI family protein (2.4 and 2.8 folds) and monothiol glutaredoxin-S2-like protein (2.3- and 2.8-fold) were upregulated common DEGs. By contrast, MACPF domain-containing protein At1g1478, protein DOWNY MILDEW RESISTANCE 6 and Fascicilin domain protein were common DEGs more than 2-fold downregulated at both 550 and 700 ppm elevated CO_2_ concentrations. These DEGs could be candidates for elevated CO_2_-mediated stress, irrespective of the imposed CO_2_ concentrations. A predicted uncharacterized protein LOC1148941 isoform X1 (2.4- and −4.9-fold) and adenine phosphoribosyl transferase 4-like protein (2.3- and −2.3-fold) were reciprocally regulated at 550 and 700 ppm elevated CO_2_ concentrations, respectively.

#### Common DEGs across different cultivars, stages and CO_2_ concentration

A predicted uncharacterized protein, LOC11489471 isoform X1 (Gene ID: XLOC-022439), was significantly regulated in K-550-15 and K-550-30 (3.9- and −2.4-fold) and between K-700-15 and K-700-30 (−2.5- and −4.9-fold). Similarly, GDSL-like lipase/acylhydrolase was commonly expressed in J-550-15 and J-550-30 (2.9- and 4-fold) and between J-700-15 and J-700-30 (2.8- and 4.3-fold).

### Hierarchical clustering of DEGs and gene network analysis

**Fig. 3:**
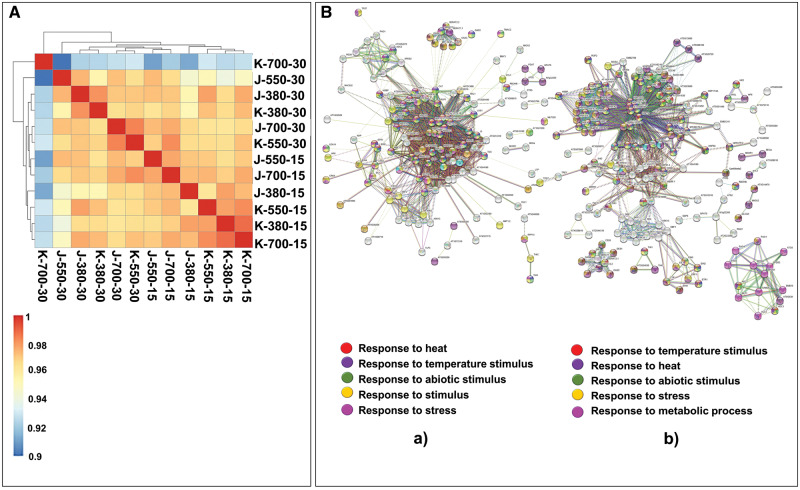
Hierarchical clustering of DEGs and gene networks. (A) Hierarchical clustering of significant DEGs across samples altered in JG 11 and KAK 2 varieties at 380 (control), 550 (stressed) and 700 ppm (stressed) of CO_2_ concentration at vegetative (15 d) and reproductive (30 d) stages. (B) Gene network analysis as observed in STRING (V.2.0) of significantly regulated DEGs in (a) JG 11 and (b) KAK 2. Functional enrichment of pathways in the networks is shown in different colors as depicted in the legend box. In KAK 2, significantly more connected nodes and edges, higher network connectivity is observed.

Hierarchical clustering analysis was performed among the DEGs significantly regulated in 12 samples (Fig. 3A). The DEGs showing similar expression patterns were clustered together. The DEGs of K-700-30 showed unique clustering pattern and did not cluster together with any of the samples studied. Prolonged exposure to stress (up to reproductive stage) at higher stress point (700 ppm) triggered more number of DEGs expressed (including exclusively expressed DEGs) in this sample (Fig. 2). To further decipher the role of significant DEGs with gene function during elevated CO_2_ concentrations, gene regulatory network analysis was performed in DEGs of two cultivars (Fig. 3B). The list of DEGs was mapped with the global Arabidopsis protein association network (STRING v.11). The significantly regulated DEGs in JG 11 (3B-a) and KAK 2 (3B-b) were analyzed separately.A larger number of gene association networks were observed to be more clustered than expected in similar sized STRING networks. In KAK 2, significantly more connected nodes and edges, higher network connectivity and average degree (edges per node), but lower average path length and number of isolated networks, were observed (Fig. 3B). Response to heat, temperature, abiotic stress stimulus and metabolic processes were the common networking clusters in both cultivars. These clusters are important for abiotic stress regulatory networks. This result denoted a possible interlink between abiotic stress regulatory networks and the elevated CO_2_ concentration-specific network.

### Expression dynamics of major TF families

TFs represent the key molecular switches orchestrating the regulation of plant growth and developmental processes. In the present study, a total of 9,687 TFs encoding genes were differentially expressed. These 9,687 TFs were categorized into at least 63 families. The top 10 largest number of TF encoding genes identified belongs to the TF families: bHLH (basic helix-loop-helix) (1072), NAC (no apical meristem, Arabidopsis transcription activation factor and cup-shaped cotyledon) (703), MYB-LIKE (673), ERF (Ethylene Responsive Factor) (557), C2H2 (513), MYB (329), WRKY (411), C3H (366), bZIP (Basic Leucine Zipper Domain) (331), B3 (313), FAR1 (FAR-RED IMPAIRED RESPONSE1) (305) and so forth. Heatmap representing differential expression profiles of some important TF families is depicted in Supplementary Fig. S2.

A large number of DEGs comprised TF families like NAC, MYB and WRKY and reaffirmed the regulation of biotic and abiotic stress signaling pathways under elevated CO_2_ concentrations (Hsu et al. 2018). A number of TF families, known to be modulating plant hormone signaling (ERFs, bZIP), were significantly represented under elevated CO_2_ concentrations.

**Fig. 4:**
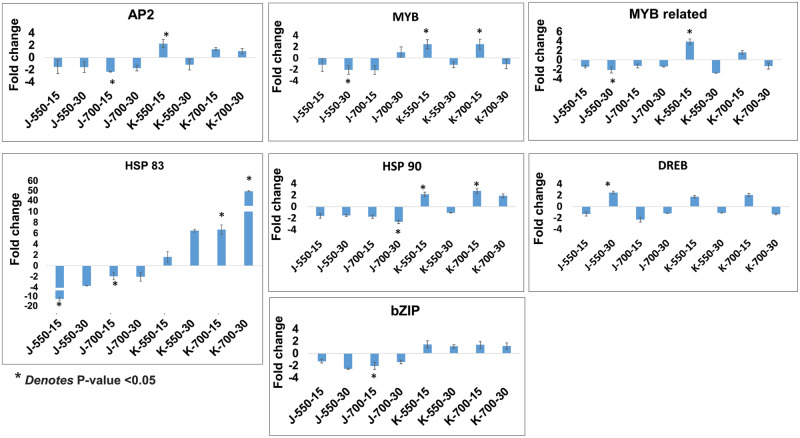
qRT-PCR expression dynamics of seven major TFs in JG 11 and KAK 2 varieties at vegetative (15 d) and reproductive (30 d) stages under 550 and 700 ppm CO_2_ concentrations, respectively. *Y*-axis denoted fold change where a minus sign denotes downregulation and value above 0 denotes upregulation. *X*-axis denoted sample IDs. Two technical replicates and three biological replicates for each sample were taken in the qRT-PCR analysis. The expression level fold changes at 550 and 700 ppm CO_2_ concentrations were compared with that obtained at 380 ppm CO_2_ concentration treatment.

Furthermore, the expression dynamics of seven important and representative TFs, which were modulated in various stress-responsive pathways, were evaluated and validated through quantitative real-time polymerase chain reaction (qRT-PCR) (Fig. 4). The in silico expression patterns of seven TFs studied were statistically significant (*P* <0.05), and the expression patterns were validated using qRT-PCR. Among them, AP2 TF showed more than 2-fold downregulation in J-700-15 (−2.33-fold) and upregulation in K-550-15 (2.23-fold). Heat shock protein 83 showed cultivar-specific differential regulation. It showed a significant downregulation in J-550-15 (−12.7-fold) and J-700-15 (−2.21-fold) and upregulation in K-700-15 (6.66-fold) and K-700-30 (49.3-fold). It also showed a wide range of differential expression with respect to elevated CO_2_ concentrations, almost 12-fold downregulation in J-550-15 to 50-fold upregulation in K-700-30. HSP 90 showed more than 2-fold downregulation in J-700-30 and more than 2-fold upregulation in both K-550-15 and K-700-15. The MYB TF gene showed more than 2-fold downregulation in J-550-30, whereas more than 2-fold upregulation in K-550-15 and K-700-15. MYB-related protein TF gene showed more than 2-fold downregulation at J-550-30 (−2.1-fold) but showed more than 3-fold upregulation at K-550-15 (3.9-fold). This showed a possible cultivar-specific differential regulation for the MYB TF family. Similarly, a bZIP family TF gene showed more than 2-fold downregulation in J-700-15 and DREB showed more than 2-fold upregulation in J-550-30. Hence it was evident that the plant stress-responsive TF families showed differential regulation under elevated CO_2_ concentrations at specific growth stage and specific level of stress imposed. This indicated the possible role of these candidate genes/TFs in response to elevated CO_2_ concentrations.

### Correlation of gene ontology and KEGG pathways with physiological alterations

Out of 19,278 significant DEGs, 18,644 were annotated using BlastX and the gene ontology (GO) terms were assigned to 11,492 genes. For these 11,492 genes, a total of 23,592 GO terms were obtained. It was seen that the GO terms for DEGs were uniformly assigned to each of the biological process (7,796), molecular function (8,878) and cellular component (6,918) categories. GO enrichment analysis of DEGs revealed that, under the biological process category, metabolic process, biological regulation and regulation of biological process were significantly represented. Among the molecular function category, catalytic activity binding and transporter activity were significantly represented. In the cellular component category, membrane, cell, cell part and organelle were the most enriched ones (Supplementary Fig. S3A).

**Fig. 5:**
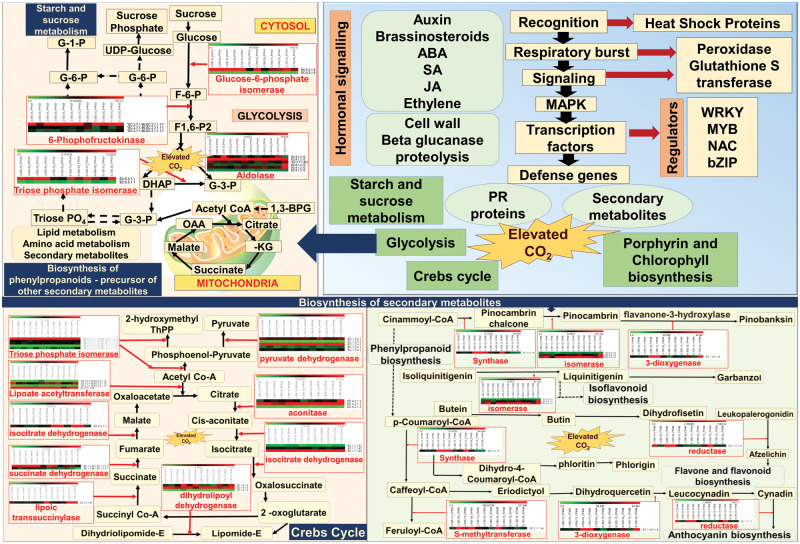
Crosstalk of major physiological pathways found altered in chickpea under elevated CO_2_ concentration. Enzymes and their expression levels in 12 different samples/conditions were shown as heat maps against respective metabolic steps in pathways.

To identify the major cross-talking pathways significantly altered under elevated CO_2_ concentrations, the pathway analysis of DEGs was carried out using the KEGG database. The DEGs represented a total of 138 pathways, which were altered under elevated CO_2_ concentrations. The enrichment analysis suggested that, under elevated CO_2_ concentrations, biosynthesis of antibiotics, purine metabolism, starch and sucrose metabolism, amino sugar and nucleotide sugar metabolism, glycolysis/neoglucogenesis, porphyrin and chlorophyll metabolism, glycine serine and threonine metabolism, citric cycle and flavonoids biosynthesis were among the most enriched pathways. The details of the KEGG pathways significantly enriched under elevated CO_2_ concentrations are listed in Supplementary Table S3 (Supplementary Fig. S3B). The major interlinked pathways with altered gene expression of the major DEGs represented through a heatmap are presented in Fig. 5. The role of elevated CO_2_ in affecting starch and sugar signaling and carbon partitioning is widely cited (Thompson et al. 2017). The findings of the present study provide further evidence, as a large number of DEGs representing major rate-limiting enzymes of glycolysis/gluconeogenesis (24 enzymes), the galactose metabolism pathway (15), starch and sucrose metabolism pathways (29), porphyrin and chlorophyll metabolism (23), the phenylpropanoid biosynthesis pathway (9) and purine metabolism (40) were significantly regulated (*P* < 0.05). For example, the enzyme EC:3.2.1.2 (saccharogen amylase) (XLOC_002641 inactive beta-amylase 9) is an important enzyme of the starch and sucrose metabolism pathway (map 00500), which was −2.62- and −2.2-fold downregulated in J-700-15 and K-550-15, respectively. The enzyme EC:2.4.1.123 (3-alpha-galactosyltransferase) (XLOC_008264 galactinol synthase), an enzyme of the galactose metabolism pathway (map 00052), was 2.33-fold upregulated in J-550-15. The enzyme EC:1.11.1.7 (lactoperoxidase) (XLOC_000819 respiratory burst oxidase-like protein) of the phenylpropanoid biosynthesis pathway (map 00940) showed 4.89-, 3.28- and 2.61-fold upregulation in J-550-15, J-700-15 and K-550-15, respectively. The enzyme EC:1.14.11.13 (2beta-dioxygenase) (XLOC_001180 gibberellin 2-beta-dioxygenase-like) of the diterpenoid biosynthesis pathway (map 00904) showed −2.75- and −2.65-fold downregulation in J-700-30 and K-550-30, respectively. The enzyme EC:3.2.2.2 (nucleosidase) (XLOC_003668 DNA-3-methyladenine glycosylase I) is an enzyme of the purine metabolism pathway (map 00230), which was 2.26-fold upregulated in J-700-15. The enzyme EC:3.6.1.15 (phosphatase) belongs to the pathway for purine metabolism (map 00230) and thiamine metabolism (map 00730). Two DEGs, the ABC transporter B family protein (XLOC_004027) and PPR containing plant-like protein (XLOC_004100), of the phosphatase enzyme group showed −2.18- and −2.04-fold downregulation in K-700-30, respectively. The enzyme EC:1.7.1.3 (reductase–NADPH) [XLOC_004657 nitrate reductase (NADH)-like protein] of the nitrogen metabolism pathway (map 00910) showed 2.01- and 2.4-fold upregulation in J-700-30 and K-700-30, respectively.The significant physiological alteration as found in the present study with respect to chlorophyll content and NBI was again depicted through alteration in porphyrin and chlorophyll metabolism, nitrogen metabolism and other secondary metabolite synthesis pathways, like the phenylpropanoid biosynthesis pathway and the diterpenoid biosynthesis pathway, as discussed above. Elevated CO_2_ concentrations are reported to affect the nutritional quality of plants (Dong et al. 2018). The presence of high amounts of folate attributes to the nutritional quality of chickpea (Wallace et al. 2016). For instance, elevated CO_2_ concentrations in the present study found altered expression patterns of several enzymes present in the folate biosynthesis pathway.

## Discussion

### Orchestrating the physiological changes and molecular alterations under elevated CO_2_ concentrations

Increases in atmospheric CO_2_ concentration were generally thought to promote plant growth since higher amounts of CO_2_ could provide more sugars to plants as energy sources. Under certain conditions, however, elevated CO_2_ concentrations may reduce plant growth by altering the primary metabolism of plants (Takatani et al. 2014). Elevated CO_2_ concentrations combined with limited nitrogen promote the progression of plant senescence, such as leaf yellowing and anthocyanin accumulation (Aoyama et al. 2014).

The present study revealed a significant increase in shoot and root length in chickpea plants of both cultivars under elevated CO_2_ concentrations. This could be attributed to enhanced vigor as the initial effect of elevated CO_2_ concentrations in plants as evidenced by a number of previous studies (Yu et al. 2017). In chickpea, a significant increase in plant height, i.e. shoot length, but a decrease in chlorophyll content was reported earlier (Saha et al. 2013) upon exposure to elevated CO_2_ concentrations. Elevated CO_2_ concentrations generally increase root growth in free-air CO_2_ enrichment (FACE) and OTC (De Graaff et al. 2006) and cause increased fine root production (Thompson et al. 2017). A meta-analysis of FACE and OTCs found a general increase in root biomass in response to elevated CO_2_ concentrations, where root length was increased more than root diameter (Nie et al. 2013).

Nodule number variation is an important stress-responsive phenomenon in chickpea as found in other stress responses also. A substantial increase in the number of nodules in both cultivars up to 550 ppm of elevated CO_2_ concentrations, as reported in the present study, was coherent with earlier studies (Leakey et al. 2009). Furthermore, higher elevated CO_2_ concentration posed a different response in KAK 2, which is a notable issue. A cultivar-specific differential response was often seen in different plant species with respect to different traits under elevated CO_2_ concentrations in various tissues (Butterly et al. 2015). Hence, this opposite trend in KAK 2 is to be explored further.

Observations on physiological parameters indicated significant alteration (4–25% decrease) in chlorophyll content of chickpea plants upon elevated CO_2_ concentrations over the ambient condition, which can be attributed to augmenting senescence. This result was also in accordance with a previous study on the effect of elevated CO_2_ concentrations in insect–plant interactions in chickpea (Sharma et al. 2016). In another study, chlorophyll content and NBI were decreased at higher of CO_2_ concentration, which was implied to the low-nitrogen content in the leaves of oilseed rape (Himanen et al. 2008).

The molecular cross talk upon elevated CO_2_ concentration is hitherto unexplored in legumes and in most of the plant species. A few exceptional studies were the specific effects of long-term exposure of elevated CO_2_ concentrations on transcriptome response of the high yielding wheat cultivar, Norin 10 (Lin et al. 2016) and transcriptome profiling of maize and elevated CO_2_ concentrations (Kolbe et al. 2019). Combined transcriptomics with morphophysiological studies explored the improved photosynthesis under elevated CO_2_ concentrations in Jatropha (Kumar et al. 2017). Hence, our present study is a pioneering effort in revealing the molecular mechanism underlying the physiological alterations as reported through transcriptomics. The effects of elevated CO_2_ concentrations in existing abiotic and biotic stresses were already documented in many crops (Palit et al. 2020). However, most of these reports were showing major physiological alterations with respect to developmental changes (shoot and root development), and amount of secondary metabolites like chlorophyll content. In the present study, expected changes have been documented in chickpea cultivars under two different growth stages—vegetative and reproductive stages—with three different CO_2_ concentrations. Here, we have combined the aforesaid physiological alteration with global gene expression changes and altered KEGG metabolic pathways (Fig. 5).

### Important DEGs and their roles in other stress responses

The RNA-Seq transcriptome data of the 12 samples revealed a number of DEGs found significantly altered under the elevated CO_2_ concentrations. Severe such DEGs have been cited earlier in similar stress conditions and other abiotic stress conditions.Respiratory burst oxidase-like protein (upregulated in tomato in elevated CO_2_ concentrations) (Cheng et al. 2009), terpene synthase 1-like isoform X1 (tomato in elevated CO_2_ concentrations and ammonium stress) (Vega-Mas et al. 2017) and MYB TF (rice in elevated CO_2_ concentrations) (ZHu et al. 2018) were a few of them. A few DEGs were earlier reported in other legume stress interactions, e.g. calmodulin-binding protein involved in Ca^(2+)^-mediated signaling in chickpea plants reported in chickpea heat and drought tolerance (Li et al. 2018). An auxin transporter was found to be regulated under drought stress in peanut (Ding et al. 2014). Sugar Transporter Protein (STP1) was involved in carbon partitioning and shoot branching in Arabidopsis (Otori et al. 2019). An increase in plant biomass especially root and shoot development under elevated CO_2_ concentrations is a widely observed phenomenon in a number of plant species including chickpea (Saha et al. 2013, Sharma et al. 2016). A number of DEGs reported here were involved in the shoot and root development processes like patatin-like phospholipase involved in root development and auxin signaling. Alteration in its expression caused lipid changes that alter cellulose content and cell elongation in Arabidopsis affecting plant cell death and defense signaling (Li et al. 2011). Another important DEG is WALLS ARE THIN 1 (WAT1)-related protein At4g83, a plant-specific protein that regulates secondary cell wall thickness of wood fibers. These findings were in accordance with the significant increase in root and shoot lengths in both cultivars of chickpea under elevated CO_2_ concentrations as documented in the present study. It is already well cited that elevated CO_2_ concentrations and consequent global warming and drought also induce the production of secondary metabolites. These are key players of plant immune responses, i.e. salicylic acid (SA) in C3 plants (Casteel et al. 2012) and terpenoid phytoalexins in C4 plants (Vaughan et al. 2016). The increased susceptibility of maize under elevated CO_2_ concentrations elevated with fungi *Fusarium verticillioides* due to the downregulation of lipoxygenase genes and attenuation of jasmonic acid and terpenoid phytoalexins production was also reported (Vaughan et al. 2016). Interestingly, in the present study, a number of DEGs involved in the plant defense mechanism were notably upregulated under elevated CO_2_ concentrations. Examples include PR proteins, an NBS-LRR domain-containing protein and pathogen aggravation regulators like volatile methyl salicylate compounds. This was in accordance with the earlier reports, e.g. putative nuclease HARBI1 involved in soybean nematode interaction (Li et al. 2014). Terpene synthase 1-like isoform X is a volatile compound related to plant defense response and fungal pathogens, which was significantly altered. It has numerous roles in the interactions of plants with their environment, such as attracting pollinators and defending the plant against pests (Falara et al. 2011). The presence of a laccase/diphenol oxidase family protein involved in lignin and flavonoids biosynthesis (Hu et al. 2018) was also found in chickpea *Fusarium* interaction. It was also involved in browning and plant disease resistance (Raju et al. 2008). These findings further relate to the existing concern of elevated CO_2_ concentrations affecting plant defense response in the changing climate scenario.

In the present study, a much higher number of DEGs (especially an exclusive set of DEGs in the case of KAK 2 at the reproductive stage) were altered at the 700 ppm elevated CO_2_ concentration than at the 550 ppm elevated CO_2_ concentration.This is probably because prolonged stress exposure (up to the reproductive stage), at higher stress point (700 ppm elevated CO_2_ concentration), triggered some specific responses in KAK 2. The effect of a stress response is often dependent on the level of stress imposed especially in the case of elevated CO_2_ concentrations in other species. For example, the growth and biomass of five tree species were found barely affected by an elevated CO_2_ concentration of 530 ppm. However, a substantial 59% increase in biomass was observed when the elevated CO_2_ concentration was increased from 350 to 700 ppm. This may be attributed to the elevated CO_2_ fertilization effect and higher nitrogen and water use efficiency (Körner et al. 2005, Zheng et al. 2018). The varied response over elevated CO_2_ concentrations was also reported in chickpea insect interactions (Sharma et al. 2016). In the present study, the exclusive set of DEGs differentially regulated at K-700-30 comprised a number of HSPs and important stress-responsive genes and TFs. For example, zinc finger AN1 domain stress-associated protein and 17.1 kDa class II HSP upregulated up to 5-fold and glycerophosphodiester phosphodiesterase GDPDL4-like downregulated up to 6-fold. These kinds of differential responses were further seen by qRT-PCR, where HSP 83 showed almost 50-fold upregulation in K-700-30. These observations again showed a probable cultivar-specific regulation in chickpea operating more pronouncedly at higher (700 ppm) elevated CO_2_ concentrations.

### Pathways involved in physiological alterations were altered at the transcriptome level

The presence of a large number of DEGs in starch sucrose metabolic pathway, glycolysis and the Krebs cycle further reaffirmed the important changes in carbon partitioning affecting metabolic changes in plants under changing concentrations of CO_2_, the C source in plants. Also, altered nitrogen metabolism, secondary metabolism-related pathways like chlorophyll metabolism and other phenylpropanoid biosynthesis pathway (nine enzymes) were in tune with our finding of altered chlorophyll content and NBI in plants. This has a greater after effect in nitrogen balance, an important aspect of legume rhizobial symbiosis, as evidenced by the alteration in legumin storage protein genes. Also, a number of enzymes and corresponding DEGs belonged to nitrogen metabolism, phenylpropanoid biosynthesis and other secondary metabolism indicated a probable reason for the alteration in chlorophyll content and NBI, which can be studied in depth in future. Interestingly, a number of DEGs found were involved in pathogen aggravation and SA signaling. For example, salicylate carboxymethyl transferase-like protein, a known volatile compound that attracts pollinators or predators forming floral scent, was upregulated (Chen et al. 2003). Furthermore, cell wall modification related genes and fungal stress-responsive genes were significantly altered in chickpea under elevated CO_2_ concentrations. For example, armadillo/beta-catenin-like repeat protein was upregulated in chickpea and *Fusarium* interaction (Gupta et al. 2010). These examples again authenticated the previous physiological studies stating altered host–pathogen interaction, especially increased pathogen aggravation (Sharma et al. 2016) in chickpea under elevated CO_2_ concentrations.

### Important stress-regulated TFs altered under elevated CO_2_ concentrations showing possible overlap with existing stress regulatory network through a cultivar, growth stage and stress-level-specific differential regulation

Interestingly, a number of TF families differentially altered in the present study both in transcriptome and qRT-PCR analyses are well-documented TFs regulating abiotic stress and form part of ABA-dependent and -independent pathways. Interlinking of elevated CO_2_ concentrations and drought and stomatal conductance is widely reported (Hsu et al. 2018) and postulated to be governed by an ABA signaling network. In the present study, out of the seven selected TFs, MYB, MYB-related family, DREB and bZIP were a few TFs whose altered expression further reflected the possible overlap between drought and elevated CO_2_ concentration regulatory network. Notably, HSP 83 showed a wide range of differential gene expression patterns. The selected HSP 90 and HSP 83 TFs belong to the heat shock protein family of HSP/chaperone network involved in multiple stress tolerance like heat and cold tolerance, and salt tolerance in plants (Deng et al. 2018). The involvement of a bZIP in ABA and CO_2_ concentration-dependent stomatal closure was reported earlier (Hsu et al. 2018).Modulation of stomatal conductance, plant biomass, raising the photosynthetic rate and enhanced root growth via auxin signaling (Niu et al. 2016) are known. MicroRNA targeting TFs like TCP, ARF and SLS in sweet potato and elevated CO_2_ interaction were recently reported (Saminathan et al. 2019). The regulations of TFs in a cultivar at a particular stress condition indicated a probable cultivar-specific and stress-level-specific response operating behind the interaction between elevated CO_2_ concentrations and chickpea. The regulatory role of the MYB and MYB-related protein group showed more than two-fold regulation under selected conditions. The role of these TFs in abiotic stress tolerance was well cited (Wu et al. 2019). It is an essential TF for drought response in chickpea (Ramalingam et al. 2015). Hence, in the present study, differential regulation of HSP 83, HSP 90, DREB, bZIP, AP2, MYB and MYB-related TFs in chickpea under elevated CO_2_ concentrations further established their overall implication on plant stress signaling. This was reported earlier in multiple stresses, proving that the complexity of elevated CO_2_ concentration and plant interaction needs case-specific attention. Furthermore, the modulation of these major TFs showed possible overlap of many abiotic and biotic stress pathways with elevated CO_2_ concentration-responsive stress, which is an important aspect of developing climate resilience.

## Conclusion

The effect of elevated CO_2_ concentrations in plants is widely acknowledged. Its widespread implication in plant physiology in the changing climate scenario has caught worldwide attention. The present comprehensive study was undertaken in chickpea, an important legume with respect to food security and high protein source, in two popular, widely cultivated cultivars under different growth stages and three different elevated CO_2_ concentrations. This study presented significant physiological alteration with respect to chlorophyll content. It also reported alteration in porphyrin and chlorophyll metabolism and other secondary metabolite synthesis pathways. A number of stress-related major TF families like HSP 83, HSP 90, AP2, MYB and MYB-related TF families showed altered expression dynamics in our qRT-PCR study. Hence, the present study will act as a model system for studying the effect of greenhouse gases in plants. Moreover, exploring the cultivar-specific, stage-specific and stress response-specific response of plant upon elevated CO_2_ concentrations and identification of key major metabolic and stress signaling pathways could help to identify the candidate gene pools and be useful in developing climate-resilient crops.

## Materials and Methods

### Plant material

The seeds of two popular chickpea cultivar cv. JG 11 (*desi* type) and KAK 2 (*kabuli* type) used for this study were procured from the Chickpea Breeding unit from Research Program—Asia of the International Crops Research Institute for the Semi-Arid Tropics (ICRISAT), Patancheru, India. The apparently healthy seeds of both cultivars were surface sterilized with 2% sodium hypochlorite and sown in the month of November 2016 (temperature 25–30°C) in 15-cm plastic pots (five seeds/pot) containing vertisol and sand (3:1) and irrigated lightly.

### Experimental design for growing chickpea plants under elevated CO_2_ concentrations

The study was conducted using three OTCs located at the Centre for Excellence in Climate Change Research for Plant Protection in ICRISAT, Patancheru, India. The OTCs are a specialized facility where a specific level of CO_2_ concentration was maintained through the emission of CO_2_ from cylinders when needed. Its CO_2_ concentration was continuously monitored by a portable CO_2_ analyzer and controlled by computer-supported regulation of inlet valves by connecting the chamber to an automated programming system (Sharma et al. 2016). Each chamber was constructed by iron structure, keeping the top or frustum open for free-air exchange for temperature and humidity reduction. The pots of each cultivar were divided into three subsets with six replications each and kept in three different environmental conditions. Three subsets were kept separately in three different OTCs, which were adjusted to three different CO_2_ states, viz. ambient (∼380 ppm) and two elevated CO_2_ concentrations (550 ± 25 and 700 ± 25 ppm). The relative humidity and temperature of OTCs were recorded automatically with the sensors fitted inside the chambers. The elevated CO_2_ concentrations were maintained up to 12 h per day throughout the experiment. Plants were assessed daily for their growth and development. The detailed experimental design is summarized in Supplementary Fig. S1.

### Physiological data acquisition

At 15 d interval up to 30 d after germination, which denoted vegetative (15 d) and reproductive (30 d) stages, plants were uprooted and data were recorded for root and shoot lengths and nodule formation. After this age, plants became lanky hence data were not taken.

### Estimation of total chlorophyll content, total flavonoids content and NBI

Total chlorophyll content, total flavonoids content and NBI were recorded once in every 15 d up to 45 d (maturity) using a phenol meter (Force A Paris, France). Total chlorophyll and flavonoids content was expressed as µg cm^−2^. Fully expanded leaf was placed between the two parts of the phenol meter, and 10 observations were recorded from each plant and three plants for each treatment were taken into consideration with necessary calibrations of the instrument.

### Statistical analysis

All data were analyzed statistically using a three-way ANOVA to test the significance of variability between the characteristics, CO_2_ concentrations, cultivars/genotypes and their interactions using the statistical program SAS 9.4 (SAS Institute Inc., Cary, NC, USA). Normality was checked for all data. The data were homogenized using arc sine transformation before performing statistical analysis. Mean separation was performed using the Student’s *t*-test, and differences were considered statistically significant when *P*-value <0.05 (*).

### Sample collection for transcriptome sequencing

Tissue samples from two biological replicates for transcriptome sequencing (RNA-Seq) were collected from three OTC subsets mentioned in section Experimental designing for growing chickpea plants under elevated CO_2_ concentrations. The shoot, leaves and apical meristem of the same set of plants (whose physiological parameters were analyzed) were harvested, quick frozen in liquid N_2_ and stored in −80°C for RNA extraction for further molecular studies and transcriptome sequencing. The overall experimental design including RNA-Seq data analysis pipeline is depicted in Fig. 1. The details of tissue harvested are given in Table 1.

### RNA isolation and QC of samples for RNA-Seq

Total RNA was isolated (typically with 100 mg tissue sample amount) following the manufacturers’ protocol of NucleoSpin^®^ RNA isolation kit by Macherey-Nagel, Germany. The quality and integrity of the RNA was primarily checked with 1.2% MOPS Agarose reducing gel followed by running in Agilent Bioanalyzer 2100. RNA samples with RNA Integrity Number or RIN value >8 were only considered for RNA-Seq sample preparation. The absolute quantification of RNA samples was done through Qubit^®^ fluorometric quantification system (Invitrogen).

### RNA-Seq libraries preparation and bioinformatics analysis

A total of 12 RNA-Seq libraries/samples (comprising high-quality filtered reads) were generated from the physiologically evaluated plant samples at two different growth stages (vegetative and reproductive) under 380, 550 and 700 ppm CO_2_ concentrations using Illumina TruSeq RNA sample preparation protocol outlined in ‘TruSeq RNA Sample Preparation Guide’ (Part no. 15008136) at the Center of Excellence in Genomics & Systems Biology (CEGSB) at ICRISAT.The sample IDs were comprised of denoting the cultivar name (JG 11 or KAK 2), age of the plant (15 or 30 d) and CO_2_ concentration imposed (380, 550 or 700 ppm), e.g. K-700-30 means 30-day-old leaf tissue of KAK 2 cultivar under 700 ppm CO_2_ concentration imposed. The samples were sequenced on Illumina HiSeq 2500 Sequencing system at Sequencing and Informatics Services Unit, CEGSB, ICRISAT. The raw reads generated from all 12 samples were subjected to quality filtering using NGS-QCBOX, an in-house parallel, automated and rapid QC pipeline for analyzing the NGS big data of Illumina platform (Katta et al. 2015) and Trimmomatic v0.35 (Bolger et al. 2014). The low-quality reads (Phred score < 30; read length < 35 bases) and reads with adapter contamination were removed to generate a set of high-quality reads termed as clean data thereafter. The complete downstream analysis was performed on these clean datasets.

### Global gene expression data: alignment and assembly

The filtered reads from all samples were aligned on the chickpea genome (Varshney et al. 2013) using Tophat v2.1.0 (Kim et al. 2013) with default parameters. The resulting alignment reads from each sample were then used to create an RABT (Reference Annotation Based Transcript) assembly using Cufflinks (v2.1.1) (Trapnell et al. 2010). The assemblies generated were then merged into one consensus assembly using ‘Cuffmerge’, which was further used to quantify transcript abundance.

### Gene expression analysis, clustering and visualization

Transcript abundance was estimated based on FPKM. Transcripts with abundance >1 FPKM in at least one sample and quantification status as ‘OK’ were considered for further analysis. After filtering the lowly expressed genes, the fold change of each gene was calculated using ‘Cuffdiff’. The DEGs with log_2_ fold change ≥2 (induced) and/or ≤−2 (repressed) and an FPKM of ≥2 for either of the sample in each pairwise comparison were considered to be significantly differentially expressed. The transcripts exhibiting a difference of at least 2-fold change with *P*-value ≤0.05 were considered to be significantly differentially expressed. Log_2_ (FPKM + 1) values of the DEGs were further subjected to K-means clustering using Pearson correlation with an optimal number of clusters set at 20 in ‘pheatmap’ of R software.

### Gene network, annotations, GO term and pathway analysis

The Gene Network analysis was performed through mapping the DEGs with the global Arabidopsis protein association network in STRING v.11, keeping the default parameters. The expressed genes were subjected to blastX (*E*-value cutoff of ≤10–5) search against the National Center for Biotechnology Information nonredundant protein database (taxon *Viridiplantae*). The Blast hits were assigned GO terms using Blast2GO v4.1.9 (Conesa et al. 2005). Further TF encoding genes were identified using blastX against PlantTFDB (Jin et al. 2014) with an *E*-value cutoff of 1E−10. As part of the pipeline, *P*-value for enrichment was calculated for each GO term represented. Only the GO terms exhibiting a corrected *P*-value of ≤0.05 were considered significantly enriched for a given set of genes. Pathway enrichment analysis was further carried out using KEGG database.

### Quantitative real-time polymerase chain reaction (qRT-PCR)

The same RNA samples subjected to RNA-Seq samples preparation were used for qRT-PCR. The total RNA isolation and QC were done as described in the earlier section. First-strand cDNA synthesis was done from 2.5 μg total RNA using a cDNA synthesis kit (SuperScript^®^ III; Invitrogen, CA, USA) following manufacturers’ instructions. qRT-PCR was performed using Applied Biosystem 7500 Real-Time PCR System (Applied Biosystems, USA) according to the manufacturers’ instructions. The qRT-PCR gene-specific primer pairs were designed using primer 3 software (Rozen and Skaletsky 2000). The qRT-PCR conditions were followed as described by Mir et al. (2014) using SYBR Green Master Mix in 96-well plates with two technical replicates and three biological replicates using in-house primer pairs and keeping glyceraldehyde-3-phosphate dehydrogenase as endogenous controls. The qRT-PCR data were compared by using the mean Ct values of three biological replicates that were normalized to the mean Ct values of the endogenous gene. The relative expression ratios were determined using the 2^−ΔΔCt^ method, and Student’s *t*-test was used to calculate significance (Livak and Schmittgen 2001); fold changes were represented graphically.

## Supplementary Data

Supplementary data are available at PCP online.

## Data availability

The sequence data have been deposited in NCBI Sequence Read archive (https://www.ncbi.nlm.nih.gov/sra) with the BioProject ID PRJNA590236.

## Supplementary Material

pcaa077_Supplementary_Data
