# Supplementary material for: Molecular and Physiological Alterations in Chickpea under Elevated CO2 Concentrations
Source: Plant Cell Physiol. 2020 Jun 5;61(8):1449–63. doi: 10.1093/pcp/pcaa077 (PMC7434580; doi:10.1093/pcp/pcaa077)
Supplement: pcaa077_Supplementary_Data [file pcaa077_supplementary_data.zip › pcaa077-Suppl_Data/pcp-2019-e-00582-File010.pdf]

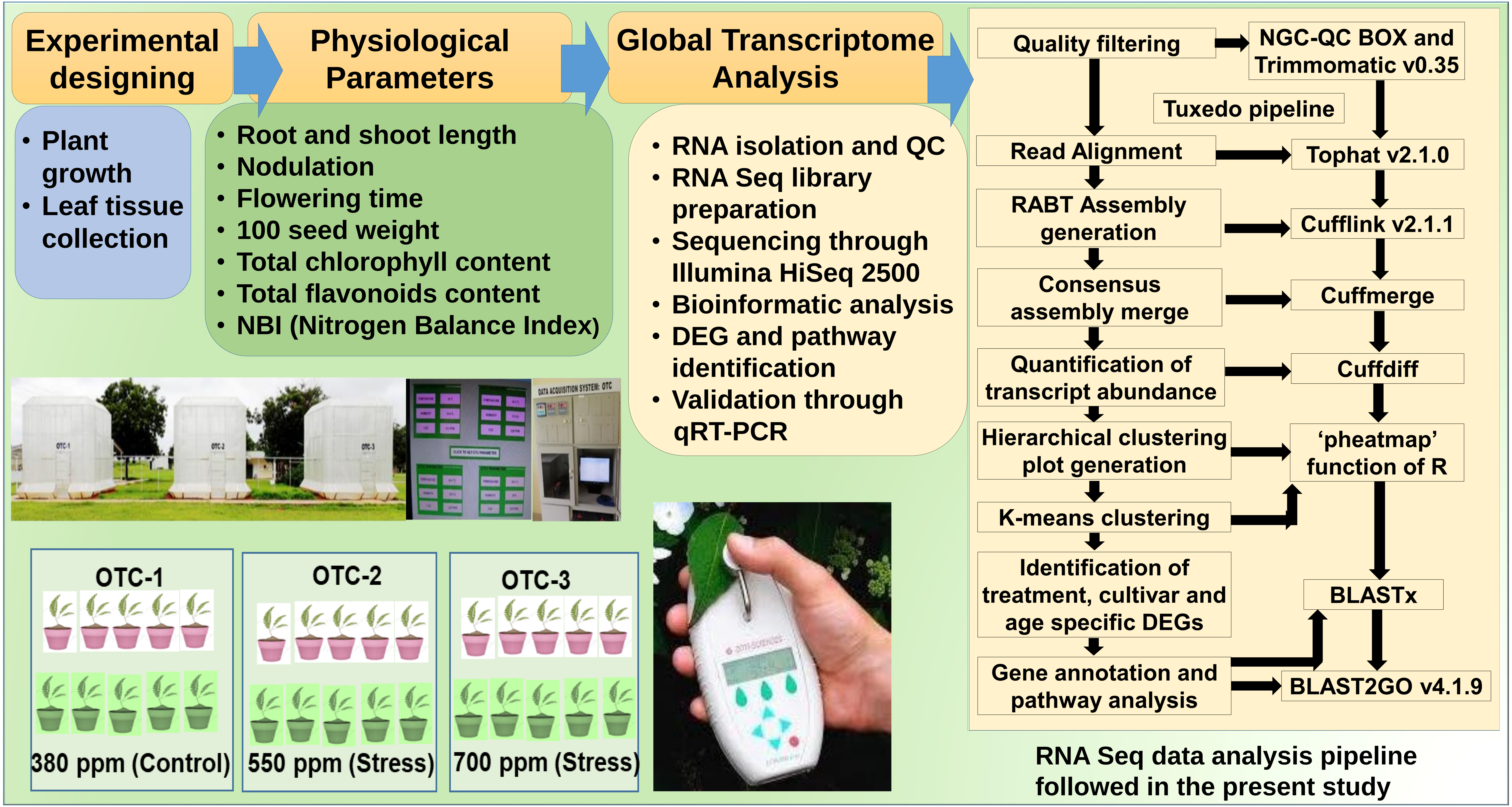

Supplementary Fig. 1 Graphical representation of the experimental design and analysis.

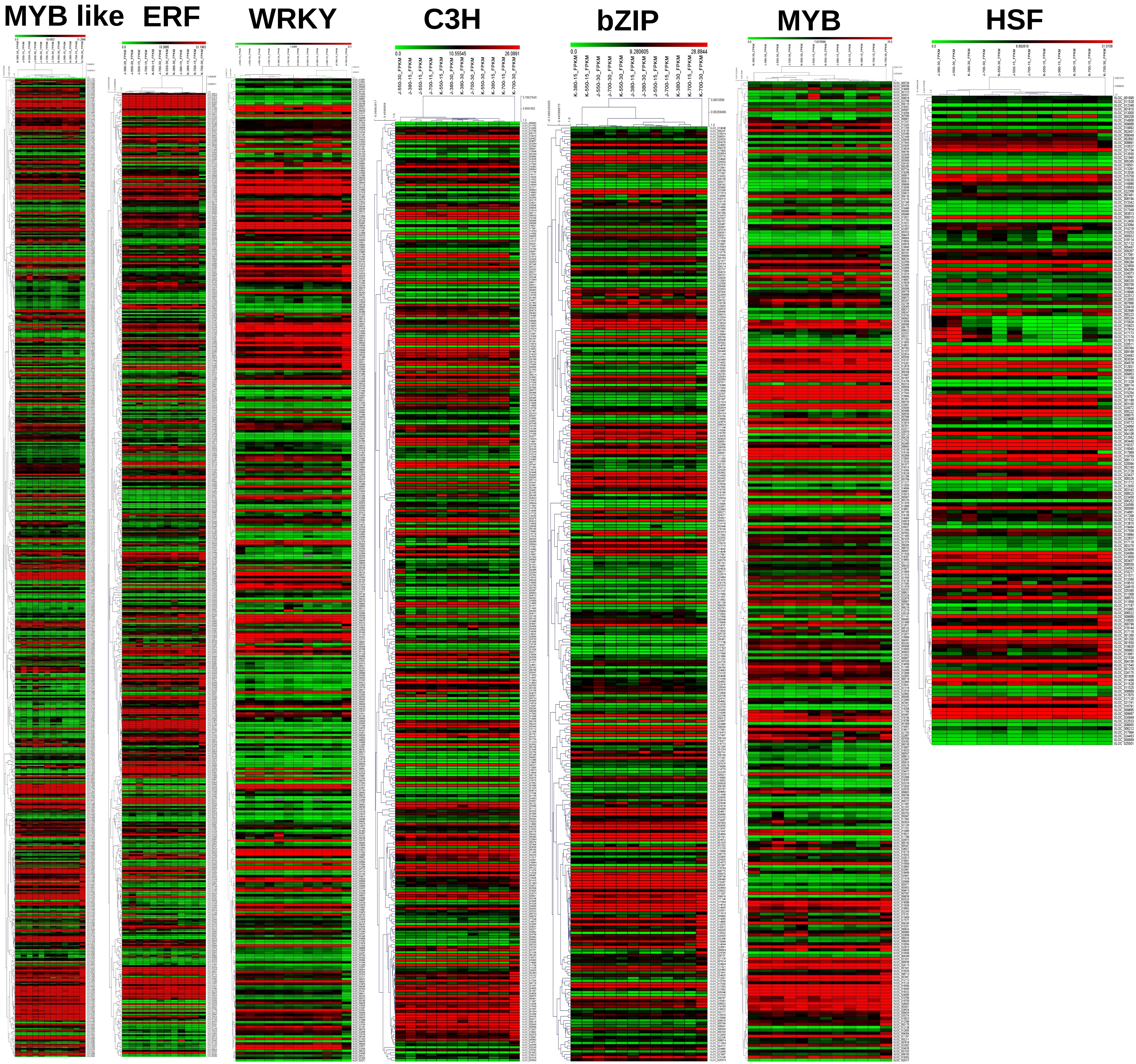

Supplementary Fig. 2 Expression profile of some important differentially expressed transcription factor (TF) gene families across samples. The unique gene identifier (prefix XLOC) is given on the right side.

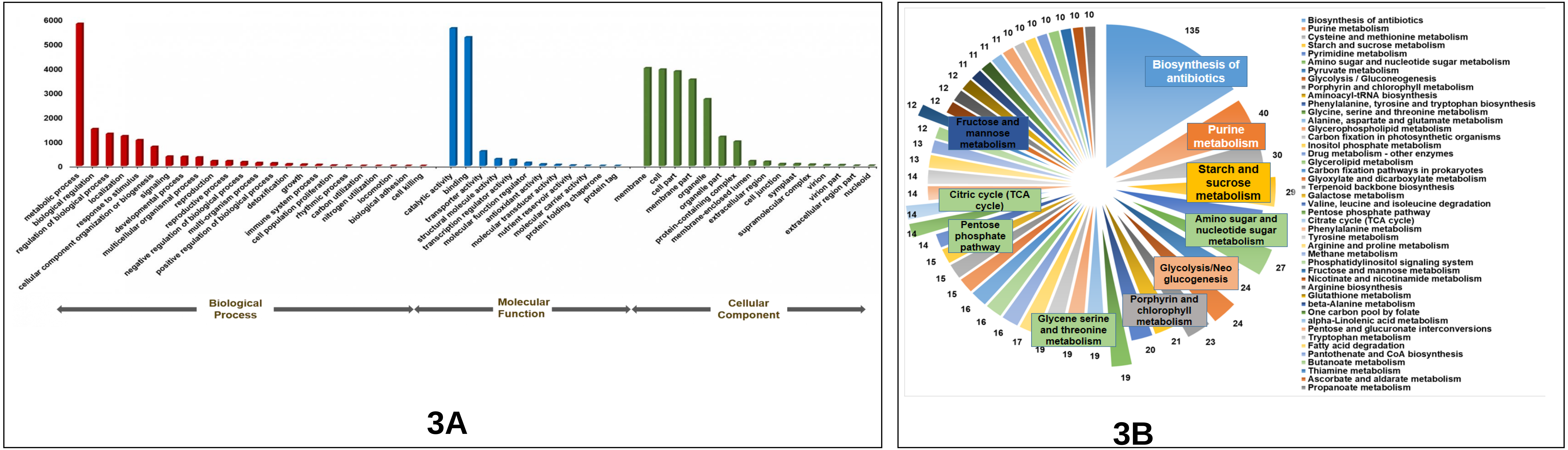

Supplementary Fig. 3 Gene Ontology and KEGG pathways in the study

3A) Gene ontology (GO) annotation of differentially expressed genes (DEGs) as altered in JG 11 (J) and KAK 2 (K) varieties at 380 ppm (control), 550 ppm (stressed) and 700 ppm (stressed) of CO_2 concentration level at vegetative (15 days) and reproductive stage (30 days). Bar graph representing GO annotations of DEGs in three categories, (a) biological processes, (b) molecular functions, and (c) cellular components.

3B) Cumulative distribution of KEGG significantly major pathways (with 10 or >10 number of genes) altered in JG 11 (J) and KAK 2 (K) varieties at 380 ppm (control), 550 ppm (stressed) and 700 ppm (stressed) of CO_2 concentration level at vegetative (15 days) and reproductive stage (30 days). The numbers on the chart indicates the total number of genes falling within each pathway
